# Cerebrospinal fluid outflow pathways in neurodegeneration: nasopharyngeal lymphatics and the ocular glymphatic-like axis

**DOI:** 10.3389/fimmu.2026.1929822

**Published:** 2026-10-01

**Authors:** Jiaqi Liu, Junzhe Chen, Shaoxiang Yuan, Yuezhong Chen, Ziyi Luo, Mingguo Xie

**Affiliations:** 1 Department of Neurosurgery, Affiliated Hospital of Zunyi Medical University, Zunyi, Guizhou, China; 2 Department of Burns and Plastic Surgery, Affiliated Hospital of Zunyi Medical University, Zunyi, Guizhou, China

**Keywords:** cerebrospinal fluid, glymphatic system, meningeal lymphatic vessels, nasopharyngeal lymphatic plexus, olfactory dysfunction

## Abstract

The glymphatic system supports cerebrospinal fluid (CSF)–interstitial fluid (ISF) exchange and has been implicated, to different extents, in the transport or clearance of amyloid-β (Aβ), tau, lactate and other solutes. Although most studies have focused on CSF influx, astrocytic aquaporin 4 (AQP4) expression, perivascular localisation and polarisation, AQP4-mediated transmembrane water transport, and perivascular exchange, brain clearance also depends on efficient extracranial drainage of waste-containing fluid. In this narrative Review, we examine this ‘outflow end’, including meningeal lymphatic vessels, the cribriform plate-olfactory-nasal mucosal route, cranial perineural spaces, the nasopharyngeal lymphatic plexus, cervical lymphatic vessels and deep cervical lymph nodes. We highlight the skull base and nasopharyngeal lymphatic plexus as potential interfaces between intracranial CSF and extracranial lymphatic drainage and evaluate the route- and cargo-specific evidence for bulk-fluid and generic-tracer transport, soluble-protein clearance, aggregated-protein handling and immune-antigen drainage. We also consider experimental strategies targeting lymphatic contractility, VEGF-C–VEGFR3 signalling, mechanical stimulation, pressure gradients, AQP4-mediated transmembrane water transport and experimentally observed light-induced pupillary modulation. Throughout, we emphasise that evidence for individual cargoes, including Aβ, tau and α-synuclein, is uneven across anatomical routes and remains predominantly preclinical or indirect. Future studies integrating high-resolution lymphatic imaging, functional biomarkers and longitudinal or interventional designs are needed to determine whether altered CSF outflow is a consequence of neurodegeneration, a contributor to disease progression or a potentially modifiable process in neurodegenerative disease.

## Introduction

The glymphatic system contributes to fluid and solute homeostasis within the central nervous system (CNS) through cerebrospinal fluid (CSF)–interstitial fluid (ISF) exchange. CSF enters the brain along periarterial spaces and exchanges with ISF within the brain parenchyma, a process associated with astrocytic endfeet and aquaporin-4 (AQP4)-mediated water transport. This system facilitates the movement of interstitial solutes, including amyloid-β (Aβ), tau and other metabolites, along paravascular pathways (1). Downstream fluid and solute transport may additionally involve meningeal lymphatic vessels and cervical lymphatic drainage routes. Although different cargoes may traverse partially overlapping anatomical interfaces, they differ in their molecular properties, aggregation states and cellular handling and should therefore not be assumed to share identical transport or clearance pathways. Beyond its mechanical and trophic functions, CSF contributes to fluid and solute homeostasis within the CNS (2, 3). Age-related reductions in CSF production and clearance may contribute to the progression of Alzheimer’s disease and other neurodegenerative disorders (4–8). More broadly, recent synthesis of mechanistic and clinical evidence has implicated glymphatic dysfunction across acute brain injury and a range of chronic CNS disorders, including stroke, traumatic brain injury, Parkinson’s disease, cerebral small-vessel disease, multiple sclerosis, idiopathic normal-pressure hydrocephalus, idiopathic intracranial hypertension, epilepsy and headache disorders (9). Understanding how CSF circulates within the brain and exits the cranial compartment is therefore fundamental to understanding brain homeostasis and neurodegeneration.

Previous studies have primarily focused on CSF influx into the brain parenchyma, AQP4-mediated water transport and factors that influence intraparenchymal clearance. However, clearance efficiency also depends on whether waste-containing fluid can exit the CNS. Accordingly, dysfunction may occur not only within the brain parenchyma but also at the ‘outflow end’, including meningeal lymphatic vessels, the skull base, nasopharyngeal lymphatics and cervical lymphatic pathways (1, 10, 11).

In recent years, there has been a growing emphasis in the field on extracranial drainage. Current evidence indicates that CSF-derived fluid and tracers can reach meningeal lymphatic vessels, the cribriform plate–olfactory–nasal mucosal route, cranial nerve-associated perineural spaces, the nasopharyngeal lymphatic plexus, and downstream cervical lymphatic vessels (1, 11, 12). The cribriform plate and the nasopharyngeal area are situated at the junction of the intracranial CSF space and the extracranial lymphatic system, potentially serving as important components for brain clearance (12). The eye-optic nerve axis may also have a similar fluid-exchange mechanism, providing a new perspective for understanding clearance relationships amongst the retina, optic nerve and CSF (13). These outflow pathways also have important neuroimmunological functions. Meningeal and cervical lymphatic networks transport CSF-derived solutes and CNS-derived antigens towards the cervical lymph nodes, thereby contributing to immune surveillance and communication between central and peripheral immune compartments. Experimental evidence further indicates that meningeal lymphatic drainage can influence the activation of brain-reactive T cells and the severity of neuroinflammatory pathology (6). Alterations in these pathways may therefore affect antigen presentation, immune-cell recruitment, inflammatory mediator distribution and the persistence or resolution of CNS inflammation.

This neuroimmune role is relevant not only to ageing and neurodegenerative disease, in which impaired meningeal lymphatic function has been associated with reduced clearance and altered immune homeostasis (7), but also to immune-mediated CNS disorders. In multiple sclerosis, drainage of CNS-derived antigens through perivascular, meningeal and cervical lymphatic pathways has been proposed to influence peripheral immune activation and disease pathophysiology (8). However, these effects are likely to depend on the anatomical compartment, integrity of the lymphatic network and stage of inflammation. Thus, the relevance of the present Review to the selected specialty lies primarily in the interface amongst CSF outflow, antigen drainage, immune regulation and neuroinflammation, rather than in a specific focus on multiple sclerosis. In this narrative Review, we examine parenchymal glymphatic exchange together with the anatomically distinct downstream pathways of CSF outflow, with a focus on meningeal lymphatic uptake, skull-base and nasopharyngeal drainage, cervical lymphatic transport, olfactory and imaging markers, and the possible disease relevance of the ocular glymphatic-like pathway.

The central unresolved issue is whether outflow-end dysfunction is simply a downstream consequence of ageing and neurodegeneration or whether it contributes to impaired clearance of Aβ, tau and other neurotoxic proteins. Resolving this question will require linking anatomical outflow routes to measurable functional impairment and disease progression. Integrated evidence from animal tracer studies, high-resolution human imaging, fluid biomarkers and longitudinal cognitive outcomes will be essential for determining whether CSF outflow failure represents a secondary phenomenon, a disease amplifier or a potentially modifiable process.

## Review methodology

This narrative Review was informed by searches of PubMed/MEDLINE and Web of Science from database inception to 31 July 2026. Search terms included combinations of “glymphatic system,” “cerebrospinal fluid outflow,” “meningeal lymphatics,” “cribriform plate,” “olfactory pathway,” “nasopharyngeal lymphatic plexus,” “cervical lymphatics,” “DTI-ALPS,” “ocular glymphatic,” and “neurodegeneration.”

Animal and human anatomical, imaging, observational, and interventional studies directly related to CNS fluid transport or lymphatic drainage were considered. Titles and abstracts were screened for relevance, followed by full-text assessment of potentially eligible studies. Peer-reviewed primary studies were prioritised, whereas reviews were used mainly for background and reference identification. Preprints were included only when no peer-reviewed version was available, and news articles were not used as primary evidence. As this was a narrative Review, no formal risk-of-bias assessment was performed; evidence was interpreted according to study design, species, directness of measurement, and evidence type.

## Conceptual framework and terminology

Brain fluid clearance involves several connected but anatomically and functionally distinct processes. Parenchymal glymphatic exchange refers primarily to CSF entry along periarterial spaces, exchange with interstitial fluid, and subsequent perivascular solute movement within the brain. This process should be distinguished from downstream CSF outflow, which may involve uptake by meningeal lymphatic vessels, egress through the cribriform plate or other skull-base foramina, drainage into nasal and nasopharyngeal lymphatics, and transport through cervical collecting lymphatic vessels to regional lymph nodes. Ocular and optic-nerve glymphatic-like movement represents a related but distinct fluid-transport process, whereas arachnoid granulations and venous pathways provide non-lymphatic routes of CSF outflow. In this Review, the term “CSF outflow dysfunction” is used only when the specific anatomical or functional component is identified. These conceptual distinctions, together with the proposed roles and principal limitations of each process or route, are summarised in **Table 1**, whilst their anatomical relationships within the broader brain-clearance network are illustrated in **Figure 1**.

**Table 1:** Conceptual framework distinguishing glymphatic exchange from CSF outflow pathways.

Process or route	Operational definition	Relevance to brain clearance	Key limitations and evidence status
Parenchymal CSF–ISF exchange	CSF enters the brain along periarterial or other perivascular routes and exchanges with interstitial fluid within the parenchyma. Astrocytic endfeet and AQP4-mediated transmembrane water transport may contribute to this process.	Supports intraparenchymal redistribution of water and soluble molecules and may facilitate transport of interstitial solutes towards downstream interfaces.	This is an intraparenchymal exchange process rather than a final extracranial outflow route. AQP4 expression, localisation, polarisation and transport function should not be treated as interchangeable measures. Representative evidence: (2, 19, 65, 67).
Perivascular fluid and solute movement	Refers to fluid and solute movement along periarterial, perivenous and other perivascular compartments before uptake by downstream drainage pathways.	Provides a potential anatomical and functional link between parenchymal exchange and meningeal, skull-base or cervical drainage routes.	Tracer movement does not necessarily demonstrate effective clearance. DTI-ALPS is an indirect, region-specific diffusion metric derived from predefined periventricular white-matter regions and does not measure CSF velocity, net flow direction or extracranial outflow. Representative evidence: (37, 38).
Meningeal lymphatic uptake	Dural and basal meningeal lymphatic vessels take up CSF-derived fluid, macromolecules and immune-related material and direct them towards extracranial lymphatic pathways.	Links intracranial fluid and antigen handling with downstream cervical lymphatic drainage and neuroimmune communication.	Uptake capacity varies by anatomical compartment; basal vessels appear more permissive than dorsal vessels in rodents. Direct quantification of their contribution to total human CSF outflow remains limited. Representative evidence: (1, 17, 32, 33, 45).
Skull-base egress through the cribriform plate and cranial foramina	CSF-derived fluid and solutes leave intracranial compartments along olfactory nerve-associated spaces and other cranial nerve or foraminal routes.	Connects subarachnoid CSF with nasal, orbital, facial and other extracranial tissues before lymphatic uptake.	The relative contribution of individual skull-base routes is uncertain and may differ substantially across species. Prominent transcribriform drainage in rodents should not be assumed to have the same quantitative role in humans. Representative evidence: (2, 11, 28, 35).
Nasal and nasopharyngeal lymphatic drainage	Nasal mucosal lymphatics and the nasopharyngeal lymphatic plexus receive CSF-derived material after skull-base egress and convey it towards cervical lymphatic vessels.	May act as a convergence interface between anterior or middle skull-base outflow routes and downstream cervical drainage.	Functional tracer drainage and experimental manipulation have been demonstrated mainly in rodents. Its quantitative contribution, age-related decline and disease specificity in humans remain unconfirmed. Representative evidence: (12, 48).
Cervical collecting lymphatic transport	Valved collecting lymphatic vessels transport CSF-derived material from skull-base, facial and nasopharyngeal networks towards superficial or deep cervical lymph nodes.	Provides downstream propulsion governed by vessel diameter, valve competence, smooth-muscle contraction and autonomic or mechanical regulation.	Current intervention evidence is predominantly preclinical. Increased tracer delivery to cervical lymph nodes does not by itself establish improved whole-brain solute clearance or clinical benefit. Representative evidence: (12, 48).
Ocular and optic-nerve glymphatic-like transport	Experimental studies suggest regulated fluid and solute movement amongst the retina, optic nerve, perioptic subarachnoid space and perivascular compartments, potentially involving AQP4-mediated transmembrane water transport.	May connect ocular tissues with CSF-facing compartments and downstream drainage pathways and may contribute to ocular solute removal in animal models.	Physiological transport must be distinguished from pathological tracer leakage or redistribution caused by lamina cribrosa disruption or abnormal pressure gradients. Direct functional validation in humans remains limited, and lamina cribrosa anatomy differs markedly across species. Representative evidence: (58, 59, 62, 69, 93).
Arachnoid granulations and venous outflow	CSF is absorbed across arachnoid villi or granulations and other interfaces into the dural venous circulation.	Represents a parallel non-lymphatic route of CSF outflow that may operate alongside lymphatic pathways.	Its relative contribution may vary with pressure, age, species and physiological state. Venous absorption should not be conflated with meningeal or extracranial lymphatic drainage. Representative evidence: (2).

AQP4, aquaporin-4; CSF, cerebrospinal fluid; DTI-ALPS, diffusion tensor image analysis along the perivascular space; ISF, interstitial fluid. DTI-ALPS, dynamic MRI, olfactory testing and ophthalmic imaging are assessment tools rather than anatomical components of CSF outflow and are therefore discussed separately in the text.

Citation note: author–year labels are provided only to identify the intended sources. Replace them with Zotero-generated numbered citations after the final reference order has been fixed.

**Figure 1:**
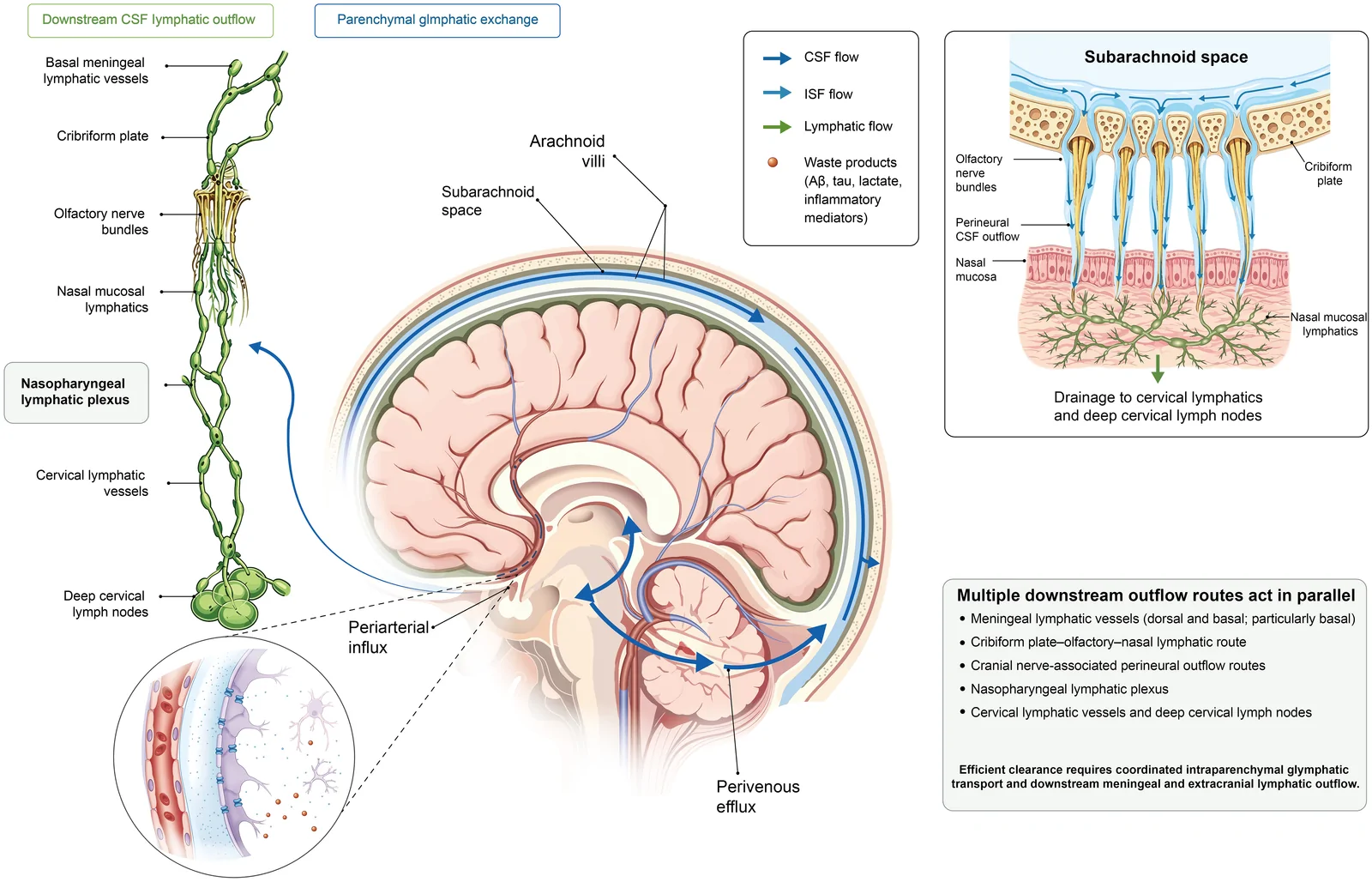
Parenchymal glymphatic exchange and downstream CSF lymphatic outflow. The image distinguishes parenchymal glymphatic exchange from anatomically downstream CSF lymphatic outflow within the broader brain-fluid clearance network. Parenchymal glymphatic exchange comprises cerebrospinal fluid (CSF) entry along periarterial spaces, exchange with interstitial fluid (ISF) within the brain parenchyma, and subsequent perivascular solute movement. After reaching CSF compartments at the brain surface, fluid and solutes may enter downstream routes, including basal meningeal lymphatics, the cribriform plate–olfactory nerve–nasal mucosal route, cranial nerve-associated perineural spaces, the nasopharyngeal lymphatic plexus, cervical lymphatic vessels and deep cervical lymph nodes. These downstream pathways are presented as components of the broader brain-fluid clearance network rather than as anatomical components of the glymphatic system itself. The inset shows CSF movement from the subarachnoid space through the cribriform plate along olfactory nerve bundles, followed by uptake into nasal mucosal lymphatics and transport towards cervical lymphatics. Efficient brain clearance therefore depends on coordination between intraparenchymal glymphatic exchange and downstream meningeal and extracranial lymphatic outflow. Blue arrows indicate CSF flow, light-blue arrows indicate ISF flow and green arrows indicate lymphatic drainage. This image is an original schematic created by the authors using Adobe Illustrator and was not adapted or reproduced from any previously published source.

## Anatomical routes of CSF and ISF outflow from the CNS

Having established the importance of the outflow end for brain clearance, it is first necessary to clarify the anatomical routes through which CSF and ISF may leave the CNS. Peripheral tissues generally depend on lymphatic circulation to remove excess fluid, macromolecules and immune cells. By contrast, the CNS was long considered to lack a conventional lymphatic system, making the route by which brain-derived metabolic waste leaves the cranial cavity a central question in neuroscience (14). The glymphatic concept partly explains CSF–ISF exchange within the brain parenchyma, yet it alone does not clarify how waste is finally transported from the cranial cavity to the surrounding areas. Consequently, meningeal lymphatic vessels, foramina at the skull base (2), perineural spaces associated with cranial nerves, and cervical lymph nodes provide an anatomical framework for understanding brain fluid and solute clearance. Anatomically, CSF and ISF outflow is unlikely to rely on a single route but may occur through multiple parallel pathways. One route comprises lymphatic vessels found in the meningeal region, especially those situated along the dural sinuses and within the base of the skull. The second category encompasses the lymphatic pathway involving the cribriform plate, olfactory nerve, and nasal mucosa. The third category pertains to the perineural spaces associated with cranial nerves and their lymphatic counterparts (15). Finally, the fourth category comprises the cervical lymphatic vessels located downstream as well as the deep cervical lymph nodes. These routes are not isolated from one another but instead form a continuous outflow network in the skull base and neck (16–19).

Initial tracer investigations offered significant insights into the pathways of CSF drainage. Upon the injection of dyes, carbon particles, India ink, or other tracers into the subarachnoid space or brain parenchyma, detectable signals were observed in the lymphatic vessels of the nasal mucosa, perineural spaces surrounding cranial nerves, and deep cervical lymph nodes. Studies that involved the removal of the dura mater from over the cribriform plate indicated that carbon particles within the CSF did not penetrate the deep cervical lymphatic vessels, implying that the cribriform plate might serve as a critical pathway for CSF drainage into the nasal and cervical lymphatic systems (20). Under physiological conditions, CSF-borne macromolecules and particulate tracers may enter the nasal submucosa. It subsequently drains into concentrated local lymphatic networks and travels through larger lymphatic vessels that connect to the nasopharynx and pharynx before arriving at the deep cervical lymph nodes. This mechanism might rely on the extensions of the subarachnoid space surrounding olfactory nerve bundles that traverse the cribriform plate (21, 22). In addition, macromolecules in the subarachnoid space can enter the perineural spaces around olfactory nerves and then diffuse through submucosal connective tissue into nasal lymphatic vessels (23). Macromolecules labelled as tracers that are introduced into the CSF can also escape via orbital pathways along the subarachnoid region encasing the optic nerve (24). Some studies further suggest that CSF drainage might occur through routes that are more direct than diffusion through the nasal mucosa (25).

Together with earlier findings (26, 27), these investigations indicate an anatomical route that facilitates the drainage of tissue fluid from the brain to lymph nodes in both normal and disease states. When tracers are injected into the cortical grey matter of rats (28), they can spread along perivascular pathways and reach compartments surrounding meningeal arteries (29). Fluids and tracers can infiltrate the meningeal layers of blood vessels, moving into areas connected to the subarachnoid space. They then proceed via the branches of the middle cerebral artery towards the circle of Willis, travelling along perivascular pathways adjacent to the naso-olfactory arteries until they reach the cribriform plate (30). From there, they can enter the nasal lymphatic vessels through the cribriform openings and eventually arrive at the deep cervical lymph nodes. The lymphoid tissues found in the head and neck predominantly consist of superficial cervical lymph nodes, deep cervical lymph nodes, and lymphoid tissue within the nasopharynx (31). CSF-derived tracers and solutes have been detected in the deep cervical lymph nodes in animal studies, supporting their role as important downstream draining lymph nodes for several CNS and CSF outflow pathways. However, they should not be regarded as the exclusive or universally established “regional lymph nodes for the brain”, and their quantitative contribution to CSF drainage in humans remains uncertain (12, 32, 33). These findings suggest that cerebrospinal fluid (CSF) does not solely flow into the venous system through arachnoid granulations; it can also leave the cranial cavity to enter the extracranial lymphatic system through the olfactory nerve and nasal mucosal lymphatics, wherein the cribriform plate serves as a crucial connection between the subarachnoid space and the nasal lymphatics.

Importantly, meningeal lymphatic vessels differ in their anatomical organisation and capacity for CSF uptake. The dorsal meningeal lymphatic vessels are primarily situated alongside the superior sagittal and transverse sinuses (34). Although functionally relevant, they have limited capacity for direct uptake of CSF tracers. In comparison, lymphatic vessels in the basal meningeal region are situated nearer to the subarachnoid space and possess button-like connections, lymphatic valves, and structural characteristics akin to pre-collecting lymphatic vessels, thereby enhancing their capacity for the absorption and transport of CSF macromolecules (33). This distinction suggests that the critical sites for CSF outflow may not lie in the traditionally emphasised dorsal dural regions but rather in the skull base and its associated lymphatic networks.

The skull base is anatomically complex and contains bony foramina, subarachnoid cisterns, vessels and cranial nerve pathways, making it an important region for the study of CSF outflow. The dorsal meningeal lymphatic vessels (dmLVs) are situated alongside the superior sagittal and transverse sinuses (34), playing a crucial role in function (32), but they show limited direct uptake and drainage of CSF tracers (35). In comparison, lymphatic vessels in the basal meningeal region are located next to the subarachnoid space, featuring blind-ended capillaries with button-like connections and valve structures that are similar to those found in pre-collecting lymphatic vessels. This configuration renders them more effective for the absorption and drainage of macromolecules from CSF (33). Contrast-enhanced MRI and fluorescence imaging further suggest that basal meningeal lymphatic vessels play a crucial role in the lymphatic clearance of macromolecules from the CSF (33). With ageing, the integrity of meningeal lymphatic vessels declines, basal meningeal lymphatics can develop lymphoedema-like changes, and CSF drainage becomes impaired (33). Simultaneously, a decrease in CSF production and turnover could create a detrimental cycle alongside dysfunction in the meningeal lymphatic system, hindering the removal of macromolecules from the CSF and possibly playing a role in the onset of Alzheimer’s disease as well as other neurodegenerative disorders (4, 5, 7, 36).

Future studies should move beyond cataloguing anatomical routes and determine the relative contribution, directionality and compensatory capacity of each outflow pathway. In particular, it remains unclear which routes dominate under physiological conditions, how their roles shift with ageing or disease, and whether findings from rodents can be translated to humans with different skull-base anatomy. Use of 3D tissue clearing, high-resolution MRI, quantitative tracer studies and cross-species comparison will be essential to resolve the spatial continuity and functional hierarchy of meningeal, cribriform, perineural and cervical lymphatic routes. Such work will help distinguish primary outflow bottlenecks from secondary or compensatory drainage pathways during neurodegeneration.

## Imaging and biomarker strategies for glymphatic and CSF outflow assessment

If anatomical studies reveal possible routes of CSF outflow, the key translational question is whether the functional status of these routes can be assessed reliably. In humans, lymphatic structures at the skull base are deep, small and difficult to interrogate directly with tracer experiments. Most human studies therefore rely on indirect imaging or functional markers of perivascular fluid dynamics and downstream drainage. In this context, DTI-ALPS, dynamic contrast-enhanced MRI, olfactory testing, cognitive assessment, and ophthalmic imaging provide complementary but biologically distinct information.

Diffusion tensor image analysis along the perivascular space (DTI-ALPS) is a diffusion MRI-derived method that calculates the ALPS index from direction-specific diffusivities in predefined projection- and association-fibre regions adjacent to the bodies of the lateral ventricles. Accordingly, the ALPS index reflects relative water diffusivity along presumed perivascular-space orientations within a restricted periventricular white-matter region; it does not directly measure CSF velocity, net flow direction, glymphatic clearance, meningeal lymphatic uptake, or extracranial CSF outflow. Although lower ALPS indices have been reported in Alzheimer’s disease and other neurological disorders, their biological interpretation may be influenced by white-matter microstructure and degeneration, fibre geometry and crossing fibres, cerebral small-vessel disease, region-of-interest definition and placement, acquisition parameters, and preprocessing procedures. DTI-ALPS should therefore be regarded as an indirect and non-specific diffusion-based marker that requires validation against direct or dynamic measures of fluid transport, rather than as a stand-alone measure of glymphatic function (37, 38).

9Olfactory dysfunction has attracted increasing attention in neuroscience, as normal olfaction can be affected by many factors and pathological conditions. Its assessment is useful for disease diagnosis, particularly because several neurodegenerative diseases are associated with olfactory impairment (39, 40). However, olfactory dysfunction is nonspecific and may also result from sinonasal disorders, including chronic rhinosinusitis, allergic rhinitis, nasal polyposis and mechanical obstruction, as well as post-infectious injury. These alternative causes should be considered when interpreting olfactory test results in relation to neurodegeneration or CSF outflow dysfunction. Abnormalities in olfactory test results have been observed in various neurological conditions, including Parkinson’s disease (PD), AD, multiple sclerosis (MS), Huntington’s disease (HD), motor neuron disease (MND) (41), as well as in several other similar disorders (37). Common olfactory tests assess the ability to identify, detect, discriminate and remember odours, as well as changes in perceived intensity when odour concentration exceeds threshold levels. However, these tests vary in reliability, the extent to which they require non-olfactory task performance and the odorants used. The Smell Identification Test from the University of Pennsylvania (UPSIT) stands as the most frequently utilised assessment. In the UPSIT, along with various other scent identification evaluations, known smells are introduced one at a time, and individuals must recognise each scent from a list of written multiple-choice alternatives. Tests for detection thresholds determine the minimum concentration of an odorant that participants can detect, typically by being compared against a blank control. The most common odour discrimination tests require participants to identify an odd stimulus amongst distractors, whereas odour memory tests assess the ability to recognise specific odours across time. Changes in perceived intensity with increasing stimulus concentration are typically assessed using rating scales and related procedures, which assign numerical values according to the relative intensity perceived by participants (42).

Future work should focus on standardising and validating these indirect assessment tools rather than treating any single metric as a direct readout of CSF outflow. Region-of-interest selection, acquisition parameters and analytical pipelines for DTI-ALPS and related imaging approaches need to be harmonised across cohorts. Imaging metrics should also be integrated with olfactory testing, ophthalmic measures, Aβ, tau and neuroinflammatory biomarkers, and longitudinal cognitive trajectories. Such multimodal frameworks will be essential for distinguishing true clearance impairment from confounding effects of white matter injury, cerebral small-vessel disease, age-related structural changes and non-glymphatic causes of olfactory or ophthalmic dysfunction.

## The nasopharyngeal and cribriform–nasal lymphatic pathways in CSF outflow

Amongst the multiple routes of CSF outflow, the nasopharyngeal lymphatic plexus deserves specific attention. Its potential relevance derives from its extracranial location and its possible role as a convergence point for CSF drainage originating from the anterior and middle portions of the skull base. CSF-derived fluid may pass through the cribriform plate along the perineural spaces surrounding the olfactory nerves and other skull-base-associated routes to reach the nasal mucosal and nasopharyngeal lymphatic networks (11, 12). Fluid and solutes may then drain through medial cervical lymphatic vessels to the deep cervical lymph nodes (12, 43). The nasopharyngeal lymphatic plexus may therefore represent a potential convergence point between skull-base CSF outflow and downstream extracranial lymphatic drainage (12). These structures form a connected drainage sequence but represent anatomically and functionally distinct compartments rather than interchangeable pathways (11, 43–47). The proposed continuity amongst the cribriform–olfactory route, nasal mucosal lymphatics, the nasopharyngeal lymphatic plexus, and downstream cervical lymphatic vessels, together with their regulatory mechanisms and potential age-related alterations, is illustrated in **Figure 2**.

**Figure 2:**
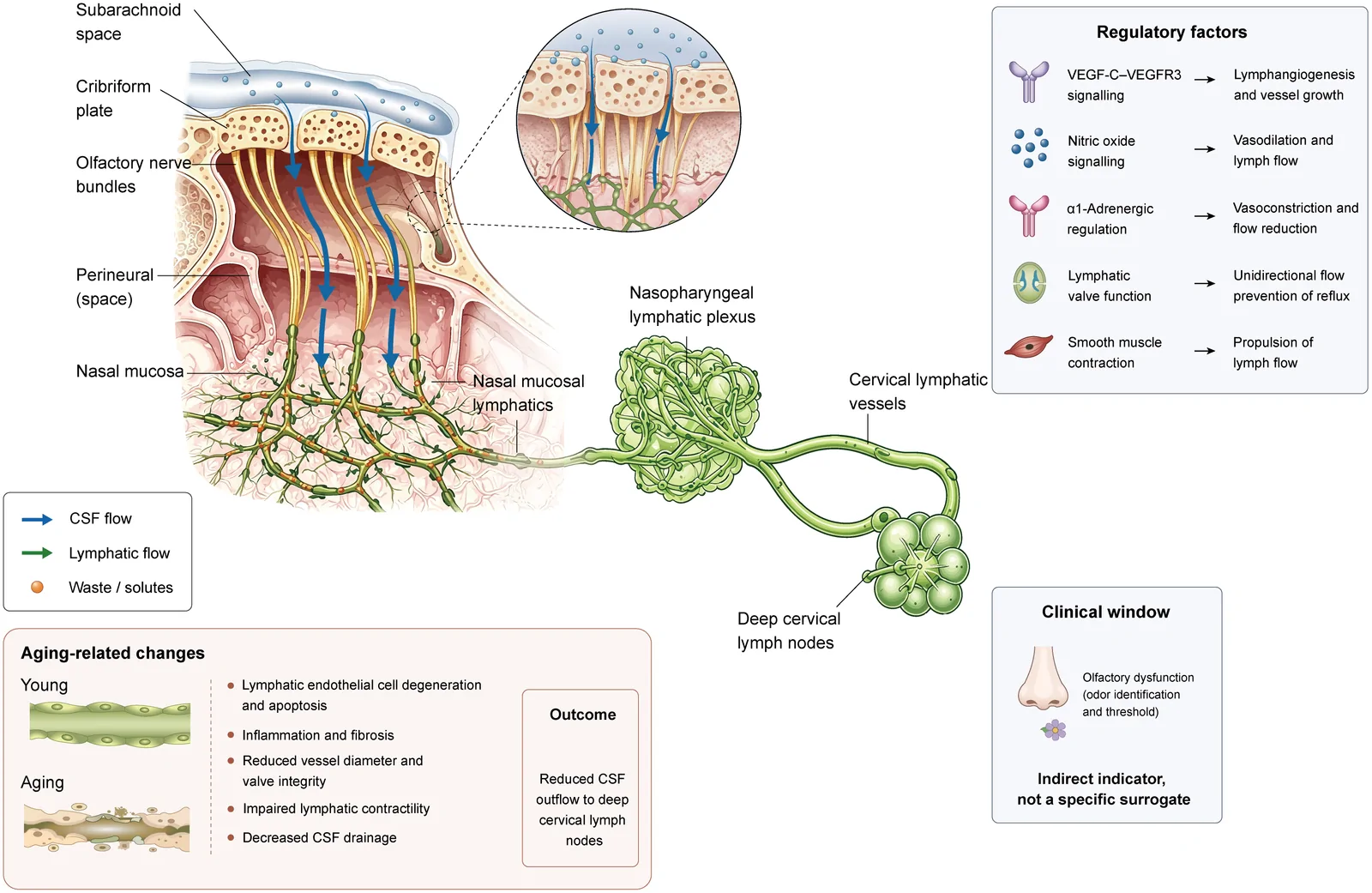
Nasopharyngeal and olfactory lymphatic drainage in CSF outflow. Cerebrospinal fluid (CSF) can drain from the subarachnoid space through the cribriform plate along olfactory nerve bundles and perineural spaces into the nasal mucosa and nasal mucosal lymphatics. CSF-derived solutes may then pass through the nasopharyngeal lymphatic plexus and cervical lymphatic vessels to reach deep cervical lymph nodes, positioning the plexus as a potential convergence point between skull-base CSF outflow and downstream cervical lymphatic drainage. The regulatory mechanisms summarised in the schematic derive from experiments performed in different anatomical compartments and should not be interpreted as regulatory properties demonstrated directly in the nasopharyngeal lymphatic plexus. Direct evidence concerning this plexus includes its short lymphangions, unusual valves, absence of smooth-muscle coverage, age-related structural regression and inflammatory changes in its lymphatic endothelial cells. Nitric oxide-mediated dilation, α1-adrenergic contraction, valve-dependent transport and smooth-muscle contractility were demonstrated in downstream medial or deep cervical collecting lymphatic vessels. VEGF-C–VEGFR3-mediated enhancement was demonstrated primarily in meningeal lymphatic vessels, whereas force-regulated facial and neck stimulation acted on superficial cervical lymphatic vessels. The schematic therefore summarises compartment-specific mechanisms operating at different levels of the broader CSF outflow network rather than mechanisms all established within the nasopharyngeal lymphatic plexus. Olfactory dysfunction may provide an indirect clinical clue to alterations along this pathway, but should not be regarded as a specific surrogate marker of cribriform, nasal, or nasopharyngeal lymphatic dysfunction.Blue arrows indicate CSF flow, green arrows indicate lymphatic flow and orange dots indicate waste products or solutes. This image is an original schematic created by the authors using Adobe Illustrator and was not adapted or reproduced from any previously published source.

In Prox1-GFP mice, the excision of the soft palate along with adjacent nasopharyngeal tissues exposes downstream lymphatic branches emanating from the nasopharyngeal lymphatic plexus on both sides, which consist of upper and lower branches. These branches merge at the posterior end of the Eustachian tube and continue towards medial deep cervical lymphatic vessels. Tracer investigations indicate that CSF can access the nasopharyngeal lymphatic plexus via lymphatic routes linked to the anterior and middle cranial fossae, subsequently travelling along medial cervical lymphatic vessels to reach deep cervical lymph nodes. Likewise, in Prox1-GFP mice and monkeys, CSF in the subarachnoid space can be drained through meningeal lymphatic vessels located in the frontal and basal areas of the skull into extracranial lymphatic vessels in the periorbital, nasal, and hard-palate regions, which then further transport fluid through superficial cervical lymphatic vessels to the submandibular lymph nodes (48). These findings suggest that the skull-base-nasopharyngeal-cervical lymphatic network is not a single pathway but consists of multiple potentially compensatory outflow routes.

The strength and nature of the evidence differ substantially across species. In rodents, tracer studies have demonstrated functional drainage from intracranial CSF spaces through skull-base and nasopharyngeal lymphatic routes, and experimental manipulation of downstream cervical lymphatics can alter tracer clearance. In non-human primates, anatomical studies support the presence of related meningeal and extracranial lymphatic connections. In humans, however, current evidence is mainly anatomical or imaging-based, and direct quantification of CSF transport through the nasopharyngeal lymphatic plexus has not yet been established. Therefore, its relative contribution to total human CSF outflow remains unknown.

Evidence for the regulation of CSF outflow along this pathway must be interpreted according to the anatomical compartment that was experimentally examined. The nasopharyngeal lymphatic plexus itself consists of short lymphangions with unusual valves and lacks smooth-muscle coverage. Therefore, the available evidence does not demonstrate intrinsic nitric oxide (NO)- or α1-adrenergic regulation of contractility within the nasopharyngeal lymphatic plexus. Direct observations in this compartment primarily concern its anatomical organisation and age-related changes. In aged mice, the nasopharyngeal lymphatic plexus undergoes structural regression, accompanied by lymphatic endothelial cell apoptosis, increased phosphorylated tau and TUNEL staining, and upregulation of type I interferon and inflammatory transcriptional programs (12).

By contrast, the evidence concerning NO signalling, α1-adrenergic signalling and smooth-muscle contractility derives from downstream medial or deep cervical collecting lymphatic vessels. These vessels have long lymphangions, semilunar valves and smooth-muscle coverage. Local application of the NO donor sodium nitroprusside or the α1-adrenergic agonist phenylephrine altered their diameter and contractile activity and, under selected dosing conditions, increased CSF transport to deep cervical lymph nodes. These findings demonstrate pharmacological modulation of downstream cervical collecting lymphatic transport rather than direct regulation of nasopharyngeal lymphatic endothelial cells. Notably, these downstream collecting lymphatic vessels retain structural integrity and pharmacological responsiveness during ageing despite regression of the upstream nasopharyngeal lymphatic plexus (12).

The anatomical targets of the other interventions are also distinct. Force-regulated mechanical stimulation was applied through the intact facial and neck skin and acted primarily on superficial cervical lymphatic vessels, increasing CSF drainage to superficial cervical lymph nodes in adult and aged mice (48). In contrast, the cited VEGF-C–VEGFR3 interventions targeted meningeal lymphatic vessels and enhanced meningeal lymphatic drainage and associated neurological outcomes in mouse models (7, 49). These findings demonstrate that several anatomically distinct components of the broader CSF outflow network can be experimentally modulated, but they do not establish that all of these regulatory mechanisms operate directly within the nasopharyngeal lymphatic plexus.

Although these findings support the functional regulatability of CSF outflow pathways, their translational status remains limited. Modulation of cervical lymphatic contractility, nitric oxide or adrenergic signalling, and mechanical stimulation has primarily been evaluated in animal tracer studies, in which increased drainage to cervical lymph nodes was observed. These studies have not established effects on human CSF outflow, neurodegenerative biomarkers, or clinical outcomes. VEGF-C–VEGFR3-based interventions have improved meningeal lymphatic function, macromolecule clearance, and selected cognitive or pathological outcomes in some aged and Alzheimer’s disease mouse models. However, these effects have not been consistently reproduced across models, and no clinical evidence currently demonstrates that VEGF-C-based lymphatic modulation improves CSF clearance or neurological outcomes in patients with neurodegenerative disease. Accordingly, these approaches should be regarded as experimental strategies for target-engagement studies rather than clinically validated therapies. Their safety, long-term effects, anatomical specificity, and dependence on disease stage remain insufficiently defined. The anatomical continuity between the cribriform–olfactory region and the nasopharyngeal lymphatic plexus provides a plausible route for CSF egress but does not establish a mechanistic relationship between CSF drainage and olfactory function. Olfactory nerve bundles traverse the cribriform plate and enter the nasal mucosa, creating an anatomical route through which CSF may pass from the subarachnoid space into nasal mucosal lymphatics and subsequently drain towards the cervical lymphatic system. However, anatomical proximity alone does not demonstrate that impaired drainage causes olfactory dysfunction or that olfactory dysfunction indicates failure of this outflow route.

Importantly, however, olfactory dysfunction is nonspecific and should not be interpreted as a biomarker or direct surrogate of CSF outflow dysfunction. Impaired olfaction may result from primary degeneration of the olfactory epithelium, olfactory bulb, or central olfactory pathways; sinonasal inflammatory or obstructive disease; ageing; smoking; viral injury; medication effects; Parkinsonian or Alzheimer-related pathology; or cognitive impairment affecting test performance. Abnormalities of the cribriform plate or nasal lymphatic drainage represent only one possible explanation. Olfactory testing should therefore be regarded as an auxiliary clinical clue that requires interpretation together with sinonasal assessment, imaging, molecular biomarkers, and longitudinal clinical findings.

Clinical studies have reported associations between olfactory function and indirect measures of brain clearance; however, these findings do not establish a specific or causal relationship with CSF outflow. The capacity for smell tends to decrease as individuals grow older (50) and is linked to cognitive abilities (51). In studies related to Alzheimer’s disease, those carrying the ϵ4 variant of apolipoprotein E (ApoE-ϵ4) may experience difficulties in identifying odours, even if their cognitive abilities remain intact (52). Furthermore, individuals exhibiting β-amyloid pathology alongside a decrease in olfactory function face an increased risk of cognitive deterioration (53, 54). In addition, cognitively normal individuals with poorer odour identification performance show lower DTI-ALPS indices, indicating reduced directional diffusivity along presumed perivascular-space regions rather than directly demonstrating reduced glymphatic clearance (55).

Evidence from isolated rapid eye movement sleep behaviour disorder (iRBD) should be interpreted separately from the AD-related findings described above. In individuals with isolated rapid eye movement sleep behaviour disorder (iRBD), lower DTI-ALPS indices have been associated with olfactory dysfunction. Olfactory impairment is also considered a risk marker for progression towards α-synucleinopathy (56). The DTI-ALPS index has been positively associated with UPSIT-40 odour-identification scores, information-processing speed, and semantic fluency in patients with iRBD. Lower DTI-ALPS indices have also been associated with atrophy of the amygdala, hippocampus, ventral diencephalon, and brainstem (57). These findings suggest an association amongst olfactory impairment, altered directional diffusivity along presumed perivascular-space regions, and neurodegenerative changes in iRBD, but do not establish obstructed CSF outflow or provide direct evidence of AD-related Aβ or tau clearance impairment.

Overall, the nasopharyngeal lymphatic plexus represents a potentially important anatomical component of the CSF outflow network. Olfactory dysfunction, by contrast, should be regarded primarily as an independent clinical phenotype associated with neurodegenerative, sinonasal or other conditions rather than as a marker of CSF outflow dysfunction. Its proposed use as an indirect indicator of cribriform or nasopharyngeal lymphatic dysfunction remains hypothetical. Future studies combining olfactory testing, DTI-ALPS, skull-base or nasopharyngeal imaging, molecular biomarkers, sinonasal assessment and longitudinal clinical outcomes are needed to determine whether reported associations reflect altered drainage, parallel neurodegenerative pathology or shared upstream factors. The quantitative contribution of the cribriform–nasal and nasopharyngeal routes to total CSF outflow in humans is unknown, and it has not been established whether the nasopharyngeal lymphatic plexus undergoes measurable age-related decline in humans or whether its dysfunction is specific to neurodegenerative disease. Sinonasal inflammation, infection, allergic disease, smoking, previous nasal or skull-base surgery, and local mucosal pathology may affect this pathway and confound its interpretation. Moreover, no validated imaging method currently provides a direct quantitative measurement of CSF drainage through the nasopharyngeal lymphatic plexus in living humans. Longitudinal studies integrating skull-base and nasopharyngeal imaging, olfactory assessment, protein biomarkers, and clinical outcomes will therefore be required. Until such evidence is available, the nasopharyngeal lymphatic plexus should be regarded as a potential component of the human CSF outflow network rather than a confirmed physiological bottleneck.

## The ocular glymphatic-like pathway: a parallel fluid-transport hypothesis

### Anatomical and tracer evidence for optic-nerve and perivascular fluid movement

The eye–optic nerve axis may represent a fluid-transport system that is related to, but distinct from, cerebral glymphatic exchange and extracranial CSF lymphatic outflow. Both the eye and the brain are relatively enclosed compartments that require precise regulation of fluid homeostasis, lack conventional lymphatic vessels within their neural parenchyma, and are vulnerable to age-related protein deposition, vascular injury and impaired solute transport (12). Fluid movement amongst the retina, optic nerve and perioptic subarachnoid space therefore provides a framework for investigating possible interactions between ocular and cerebral clearance processes. However, the ocular glymphatic-like pathway should be regarded as a parallel hypothesis rather than an established component of whole-brain CSF or protein clearance.

Experimental studies support the presence of fluid movement along perivascular and peri-axonal spaces of the optic nerve. CSF-derived tracers can enter the optic nerve from the surrounding subarachnoid space and move along perivascular pathways (58, 59). Following the administration of India ink into the subarachnoid space, tracer particles have been detected within perivascular spaces and collagen-associated compartments of the optic nerve (60, 61). Intravital tracer experiments involving injection into the cisterna magna have similarly demonstrated movement of CSF-derived tracers along the optic nerve in rodents (58, 59).

Transport may also occur in the opposite direction. Experimental tracers introduced into the retina or vitreous can move towards the optic nerve and subsequently reach extracranial drainage sites. The detection of ocularly or intracranially administered tracers in cervical lymph nodes suggests that ocular and cerebral fluid-transport pathways may ultimately interact with common downstream lymphatic structures (62). These observations support bidirectional fluid and solute movement amongst the retina, optic nerve, subarachnoid space, and cervical lymphatic system. The use of common downstream cervical lymphatic structures does not, by itself, establish a quantitatively important contribution of ocular transport to cerebral CSF clearance.

However, most of this evidence is derived from animal tracer studies. These experiments demonstrate anatomical continuity and tracer movement but do not establish the physiological magnitude, dominant direction, or quantitative contribution of this pathway under normal human conditions. Direct functional evidence for ocular–optic nerve fluid transport in living humans remains limited.

## Differences between cerebral glymphatic transport and ocular glymphatic-like transport

Although cerebral and ocular fluid-transport systems share several features, the ocular pathway should not be regarded as a direct anatomical or mechanistic replica of the cerebral glymphatic system. In the brain, AQP4 is expressed by astrocytes and is preferentially localised and polarised at their perivascular endfeet (63–65). In the eye, AQP4 is expressed by retinal Müller cells and optic-nerve astrocytes, indicating a potential role in local water homeostasis and perivascular fluid movement (66). However, evidence of AQP4 expression or cellular localisation does not, by itself, demonstrate preserved perivascular polarisation or functional AQP4-mediated transmembrane water transport.

Total AQP4 expression, cellular localisation, perivascular polarisation and functional AQP4-mediated transmembrane water transport represent distinct levels of biological evidence and should not be treated as interchangeable indicators of clearance function. Experimental studies suggest that AQP4-mediated water transport contributes to ocular fluid and Aβ movement along the optic nerve (62), but these functional observations should be distinguished from evidence based solely on AQP4 abundance or anatomical distribution.

A major anatomical difference is the lamina cribrosa, which forms an interface between the intraocular and intracranial pressure compartments. This structure permits the passage of retinal ganglion cell axons and blood vessels whilst contributing to the regulation of fluid and pressure transmission between the eye and optic nerve. Its composition also differs across species. In humans, the lamina cribrosa contains prominent collagenous and elastic connective-tissue beams, whereas the corresponding region in rodents is more strongly supported by astrocytic structures. These differences limit direct extrapolation of ocular tracer findings from rodents to humans.

The lamina cribrosa may also restrict the passage of extracellular tracers. Experimental studies suggest that tracers introduced into the vitreous do not freely cross the laminar region and may require neuronal uptake and axonal transport to enter the optic nerve. Similarly, the lamina cribrosa can limit the movement of CSF-derived tracers from the perioptic subarachnoid space towards the retina (62, 67, 68). Ocular glymphatic-like transport is therefore shaped by anatomical barriers, pressure gradients, axonal transport, and glial water regulation that differ substantially from those operating in the cerebral parenchyma.

For these reasons, the term “ocular glymphatic-like pathway” is used in this Review to describe a proposed fluid-transport system that shares selected features with cerebral glymphatic exchange but has distinct anatomical, regulatory, and physiological constraints. Within this framework, regulated physiological transport should be distinguished from pathological tracer redistribution. Under physiological conditions, ocular glymphatic-like transport refers to fluid and solute movement amongst the retina, optic nerve, perioptic subarachnoid space, and downstream drainage compartments, potentially involving AQP4-mediated transmembrane water transport. By contrast, structural disruption of the lamina cribrosa or abnormal translaminar pressure gradients may cause non-specific tracer leakage or redistribution. Increased tracer penetration under these pathological conditions should therefore not be interpreted as enhanced clearance. Current evidence for both scenarios is derived predominantly from animal tracer studies, whereas direct functional validation in humans remains limited. This distinction is particularly important when interpreting tracer findings in glaucoma. **Figure 3** summarises the proposed ocular glymphatic-like pathway and illustrates regulated physiological fluid transport and pathological tracer leakage or redistribution associated with lamina cribrosa disruption.

**Figure 3:**
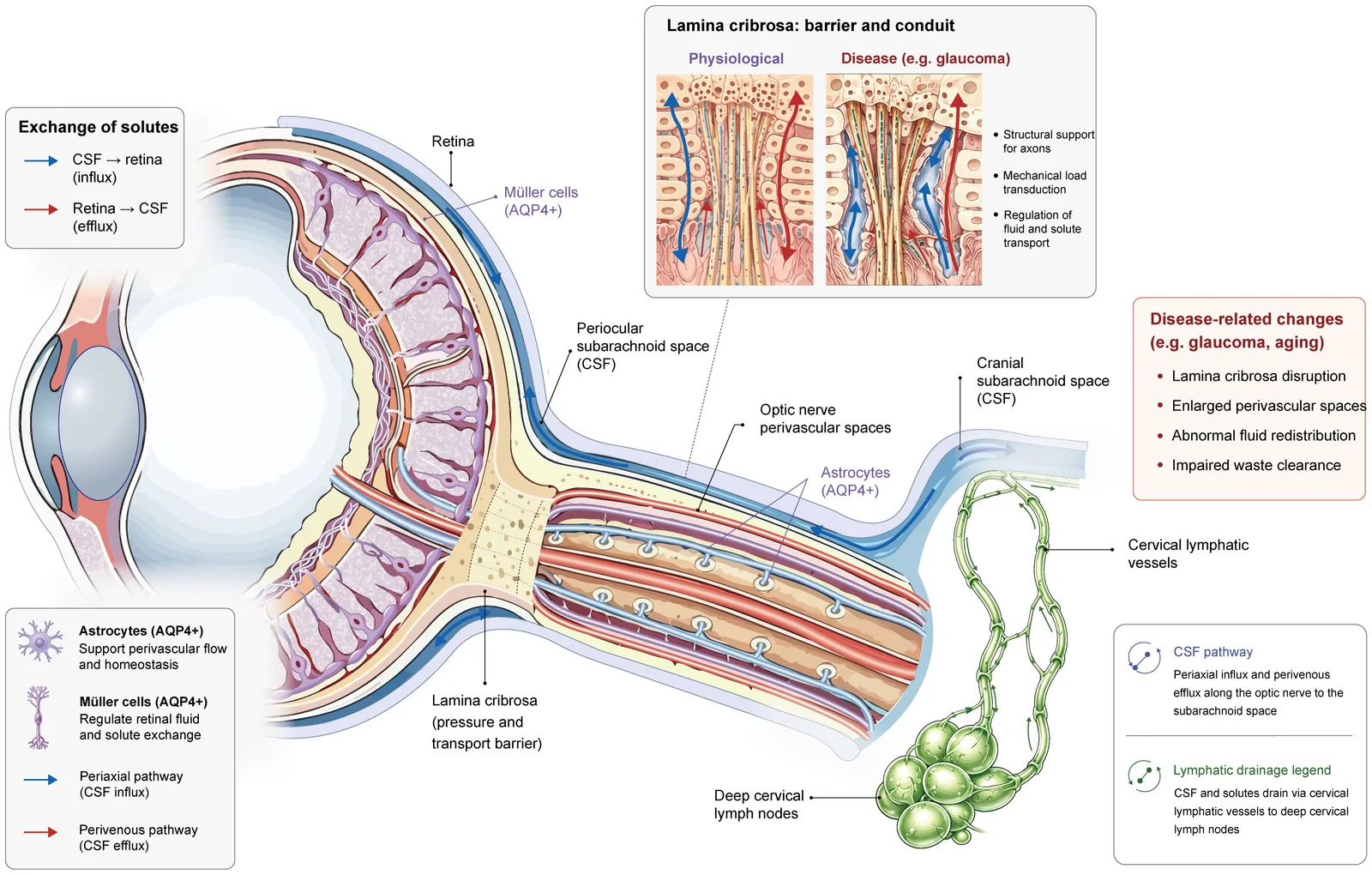
Ocular glymphatic-like pathway and optic nerve clearance. The eye–optic nerve axis may represent a glymphatic-like extension of the CNS clearance network. Cerebrospinal fluid (CSF) can move from the cranial and periocular subarachnoid spaces along perivascular spaces of the optic nerve towards the retina, whereas solutes from the retina and optic nerve may be cleared in the opposite direction towards the CSF compartment and downstream cervical lymphatic vessels. Müller cells in the retina and astrocytes in the optic nerve express aquaporin 4 (AQP4), supporting local water movement, perivascular fluid exchange and tissue homeostasis. The lamina cribrosa acts as both a pressure barrier and a transport-regulating structure between the intraocular and intracranial compartments. Under physiological conditions, this region helps maintain controlled fluid and solute exchange; in disease states such as glaucoma, disruption of the lamina cribrosa and enlargement of perivascular spaces may lead to abnormal fluid redistribution rather than effective clearance. Ageing and glaucoma-related changes may therefore impair waste removal from the retina and optic nerve and contribute to ocular neurodegeneration. CSF-derived solutes can ultimately drain through cervical lymphatic vessels to deep cervical lymph nodes. Blue arrows indicate CSF influx from the subarachnoid space towards the retina, red arrows indicate retinal or optic nerve solute efflux towards CSF, and green arrows indicate lymphatic drainage to cervical lymph nodes. This image is an original schematic created by the authors using Adobe Illustrator and was not adapted or reproduced from any previously published source.

## Evidence in glaucoma

Glaucoma provides the most extensively studied disease context for ocular glymphatic-like dysfunction. Reduced entry of CSF-derived tracers into the optic nerve has been reported in experimental and clinical studies of glaucoma, suggesting that altered perioptic fluid movement may accompany glaucomatous optic-nerve injury (58). At the same time, disruption of the lamina cribrosa may permit abnormal movement of extracellular tracers into the optic nerve, particularly when structural defects compromise its barrier function (68).

These apparently opposing findings indicate that tracer movement in glaucoma cannot be interpreted simply as increased or decreased clearance. Reduced tracer influx may reflect impaired perioptic transport, whereas increased or redistributed tracer signal may result from lamina cribrosa disruption, pathological leakage, or enlargement of perivascular spaces. Therefore, greater tracer penetration in glaucomatous tissue should not be regarded as evidence of improved waste removal.

Ageing and glaucoma may also influence ocular fluid transport through different mechanisms. Physiological ageing appears to reduce both anterograde and retrograde transport along the optic nerve. In glaucoma, overlapping distributions of anterograde and retrograde tracers may instead reflect abnormal fluid redistribution caused by altered pressure gradients, laminar barrier dysfunction, and perivascular-space enlargement (69).

Vascular and extracellular-matrix alterations may further contribute to disturbed fluid movement. Patients with glaucoma may exhibit capillary loss and vascular abnormalities in the anterior optic nerve (70). Experimental glaucoma models demonstrate enlargement of optic-nerve perivascular spaces, basement-membrane remodelling, and increased fibrotic collagen deposition (69). Such changes may impair nutrient delivery, disturb fluid exchange, and promote the accumulation of potentially harmful metabolites.

The translaminar pressure gradient is another important determinant of optic-nerve physiology. Lowering intraocular pressure delays the onset and progression of primary open-angle glaucoma (71), but there is currently no evidence that intraocular-pressure reduction directly restores ocular glymphatic-like clearance. Accordingly, established glaucoma treatments should not be reinterpreted as glymphatic interventions without direct measurements of fluid or solute transport.

Although glaucoma and neurodegenerative diseases share certain features, including ageing, vascular dysfunction, axonal injury and protein accumulation, findings from glaucoma should not be directly extrapolated to Alzheimer’s disease or interpreted as evidence of cerebral CSF outflow dysfunction.

## Evidence from Alzheimer’s disease and other neurodegenerative conditions

Aβ is a major pathological component of cerebral amyloid plaques and has also been detected in the retina in experimental models and in patients with Alzheimer’s disease (72, 73). These observations have raised the possibility that the retina may provide an accessible peripheral window into processes associated with cerebral protein accumulation.

Experimental studies indicate that Aβ introduced into the rodent eye can be transported along the optic nerve through an AQP4-associated pathway (62). However, these findings do not establish that impaired ocular glymphatic-like transport is a cause of cerebral Aβ accumulation or Alzheimer’s disease. Retinal Aβ deposition may reflect local production, systemic or cerebral pathology, vascular dysfunction, age-related changes, or impaired local cellular clearance in addition to altered fluid transport.

Ageing reduces glymphatic transport and the clearance of Aβ, tau, and α-synuclein within the brain (74–78). Human physiological studies have shown that pupil diameter and pupillary light responses change with ageing; however, these studies did not directly assess ocular fluid transport, Aβ clearance or ocular glymphatic-like function. Whether age-related changes in pupillary responsiveness influence ocular fluid homeostasis therefore remains uncertain, and they should not be interpreted as evidence of impaired cerebral clearance.

Aβ-containing deposits have also been identified in age-related macular degeneration (79–81). This provides evidence of shared protein-deposition biology but does not demonstrate that age-related macular degeneration is caused by ocular glymphatic-like dysfunction or that its mechanisms are equivalent to those of Alzheimer’s disease. The relationship amongst retinal Aβ deposition, ocular fluid transport, and cerebral neurodegeneration should therefore be regarded as hypothesis-generating.

Ischaemic optic neuropathy provides another example of disease-associated alteration in optic-nerve fluid dynamics. Acute ischaemia can modify CSF movement, promote optic-nerve oedema, impair axonal transport, and enhance neuroinflammation (82–84). In cerebral ischaemia, excessive CSF influx may contribute to acute tissue swelling (85). These observations suggest that fluid transport may become maladaptive during acute vascular injury. However, ischaemic optic neuropathy should be distinguished from chronic neurodegenerative disorders, and evidence from acute ischaemia should not be directly extrapolated to Alzheimer’s disease, glaucoma, or age-related retinal disease.

Overall, current evidence supports the ocular glymphatic-like pathway as a related or parallel fluid-transport hypothesis involving the retina, optic nerve and perioptic subarachnoid space. It does not establish this pathway as a functional component of whole-brain CSF clearance, demonstrate that ocular glymphatic-like dysfunction directly contributes to cerebral protein accumulation, or validate ocular measurements as specific biomarkers of cerebral CSF outflow.

## Therapeutic implications and limitations

The ocular glymphatic-like pathway currently represents an experimental framework rather than a validated therapeutic target. Proposed strategies include preservation of the lamina cribrosa and translaminar pressure gradient, modulation of AQP4-associated water transport, control of intraocular pressure, and regulation of light-induced pupillary responses.

The evidential status of these approaches differs substantially. Intraocular-pressure reduction has demonstrated clinical benefit in glaucoma, but has not been shown to restore ocular glymphatic-like clearance. By contrast, AQP4 modulation and light-induced pupillary regulation have been investigated mainly in animal models, without established effects on human clearance, neurodegenerative biomarkers, or clinical outcomes. Safety and disease-stage dependence remain largely undefined.

Evidence that light-induced pupillary responses modulate ocular glymphatic-like transport currently comes specifically from rodent tracer experiments. In these studies, light-induced pupillary constriction enhanced anterograde ocular tracer movement, whereas atropine or increased intracranial pressure reduced this transport (62). Human physiological studies have established that pupil diameter and pupillary light responses vary with ambient illumination, arousal or autonomic state, accommodative activity and ageing. However, these human studies did not directly measure ocular tracer movement, fluid clearance, Aβ transport or ocular glymphatic-like function. Moreover, no clinical disease study has demonstrated that light stimulation or manipulation of pupillary responses improves ocular or cerebral protein clearance or modifies clinical outcomes in glaucoma, age-related macular degeneration or Alzheimer’s disease. Light-induced pupillary modulation should therefore be regarded as a preclinical mechanistic observation and a hypothesis-generating translational strategy rather than an established clinical intervention.

AQP4 modulation may also have context-dependent effects. In experimental ischaemic optic neuropathy, the AQP4 inhibitor TGN-020 reduced abnormal CSF accumulation and preserved retinal and optic-nerve structure and function (83). This finding suggests that temporary inhibition of AQP4-mediated water movement may be protective during acute oedema. It does not imply that long-term AQP4 inhibition would enhance physiological clearance. Depending on disease stage and barrier integrity, increasing or decreasing AQP4-associated transport could have different and potentially opposing consequences.

Similarly, interventions targeting intraocular pressure or translaminar pressure gradients may protect optic-nerve structure without necessarily restoring ocular glymphatic-like transport. Therapeutic studies should therefore distinguish conventional neuroprotective or pressure-lowering effects from direct modification of fluid clearance.

Clinical translation is also limited by the absence of validated methods for quantifying ocular glymphatic-like transport in living humans. Optical coherence tomography and optical coherence tomography angiography can characterise retinal and optic-nerve structure and microvasculature but do not directly measure fluid clearance. Dynamic MRI, diffusion MRI, and SPECT/CT may provide indirect information on perioptic CSF dynamics or tracer distribution (86–88), but their interpretation is influenced by vascular permeability, tissue diffusion, injection route, imaging timing, and structural barrier disruption.

Future studies should therefore establish whether ocular glymphatic-like transport can be directly and reproducibly measured in humans, distinguish physiological clearance from pathological leakage, and determine whether ocular measurements provide information beyond established ophthalmic and neurological biomarkers. Interventional studies should demonstrate not only altered tracer movement but also target engagement, improved solute clearance, preservation of neural structure, and clinically meaningful visual or neurological outcomes.

Overall, the eye–optic nerve axis provides a potentially accessible model for studying fluid movement and shared degenerative processes between the eye and brain. However, its physiological contribution in humans and its relevance to cerebral neurodegeneration remain incompletely defined.

## Neuroimmune consequences of meningeal and cervical lymphatic drainage

Meningeal and cervical lymphatic pathways should not be viewed solely as routes for fluid and metabolic-waste removal. CSF-borne CNS antigens and soluble inflammatory mediators can be sampled at meningeal immune interfaces, whilst macromolecules and some immune cells can drain towards cervical lymph nodes. At the dural sinuses, CSF-derived antigens can be captured by local macrophages and dendritic cells and presented to patrolling T cells, supporting immune surveillance at the brain borders (89). Meningeal lymphatic vessels also facilitate the drainage of CSF macromolecules and the CCR7-dependent migration of meningeal immune cells, particularly T cells, towards cervical lymph nodes (6). Within these nodes, resident and migratory antigen-presenting cells integrate antigen availability with costimulatory, inhibitory and cytokine signals to shape peripheral immune responses. However, the relative contributions of free antigen drainage and antigen-bearing cell migration, and of individual meningeal, cribriform and cervical routes, remain incompletely defined.

Antigen drainage does not predetermine an inflammatory or tolerogenic outcome. Whether steady-state drainage of CNS-derived antigens preferentially supports immune surveillance, regulatory responses or peripheral tolerance has not been established for individual anatomical routes. During infection, tissue injury or autoimmunity, activated antigen-presenting cells and inflammatory signals may instead promote effector T-cell responses. In experimental Toxoplasma gondii infection, surgical interruption of lymphatic drainage to the deep cervical lymph nodes reduced dendritic-cell and antigen-specific CD4+ and CD8+ T-cell responses in these nodes but did not impair parasite control in the brain, indicating that lymphatic-dependent immune activation does not necessarily determine disease outcome (90). Conversely, ablation of meningeal lymphatics in experimental autoimmune encephalomyelitis reduced the inflammatory response of brain-reactive T cells and ameliorated disease pathology (6). These findings demonstrate that lymphatic drainage can modulate adaptive immune activation, but its consequences depend on the disease context.

Immune-cell trafficking should also be distinguished according to direction and compartment. CCR7-dependent migration from the meninges to cervical lymph nodes represents lymphatic egress, whereas recruitment of activated peripheral T cells and myeloid cells into the CNS occurs through vascular and brain-border interfaces. These processes may become functionally coupled through antigen presentation, lymph-node activation and subsequent immune-cell recirculation, but they are not anatomically interchangeable. Similarly, cytokines and chemokines present in CSF may reach meningeal or cervical lymph-node compartments and influence local signalling; however, direct quantitative evidence for the route-specific transport and biological activity of individual cytokines remains limited.

The immunological consequences may also differ between neurodegenerative and immune-mediated CNS diseases. In ageing models, meningeal lymphatic dysfunction can promote excessive microglial IL-6-related signalling and associated synaptic abnormalities (51). In 5xFAD mice, ablation of meningeal lymphatic vessels exacerbated microgliosis, neurovascular dysfunction and the adverse effects of impaired Aβ removal during passive anti-Aβ immunotherapy (91). These studies indicate that lymphatic dysfunction can influence microglial and inflammatory responses in addition to altering fluid or protein transport. By contrast, in autoimmune or infectious disease, lymphatic drainage may enhance antigen delivery to cervical lymph nodes and strengthen antigen-specific T-cell responses whose consequences may be pathogenic, protective or insufficient to alter disease control, depending on antigen identity, antigen-presenting-cell state and disease stage.

Enhanced lymphatic drainage should therefore not automatically be equated with beneficial waste clearance. Its effects depend on the transported cargo, anatomical route, inflammatory context and timing of intervention. Studies evaluating lymphatic modulation should assess not only tracer or protein clearance but also antigen presentation in cervical lymph nodes, antigen-presenting-cell activation, effector and regulatory T-cell responses, immune-cell trafficking, cytokine profiles and microglial states.

## Future perspectives

Future research should move beyond demonstrating the anatomical existence of CSF outflow routes and determine their quantitative, functional, and disease-specific relevance. Progress will require coordinated research across six priorities: (1) standardised definitions and biomarkers; (2) human imaging and functional validation; (3) longitudinal studies of causality; (4) interventional target-engagement studies; (5) safety considerations; and (6) cross-species translational challenges. **Figure 4** presents an integrated model in which dysfunction across meningeal, cribriform–olfactory, nasopharyngeal, cervical, and ocular outflow pathways may interact during ageing and neurodegeneration.

**Figure 4:**
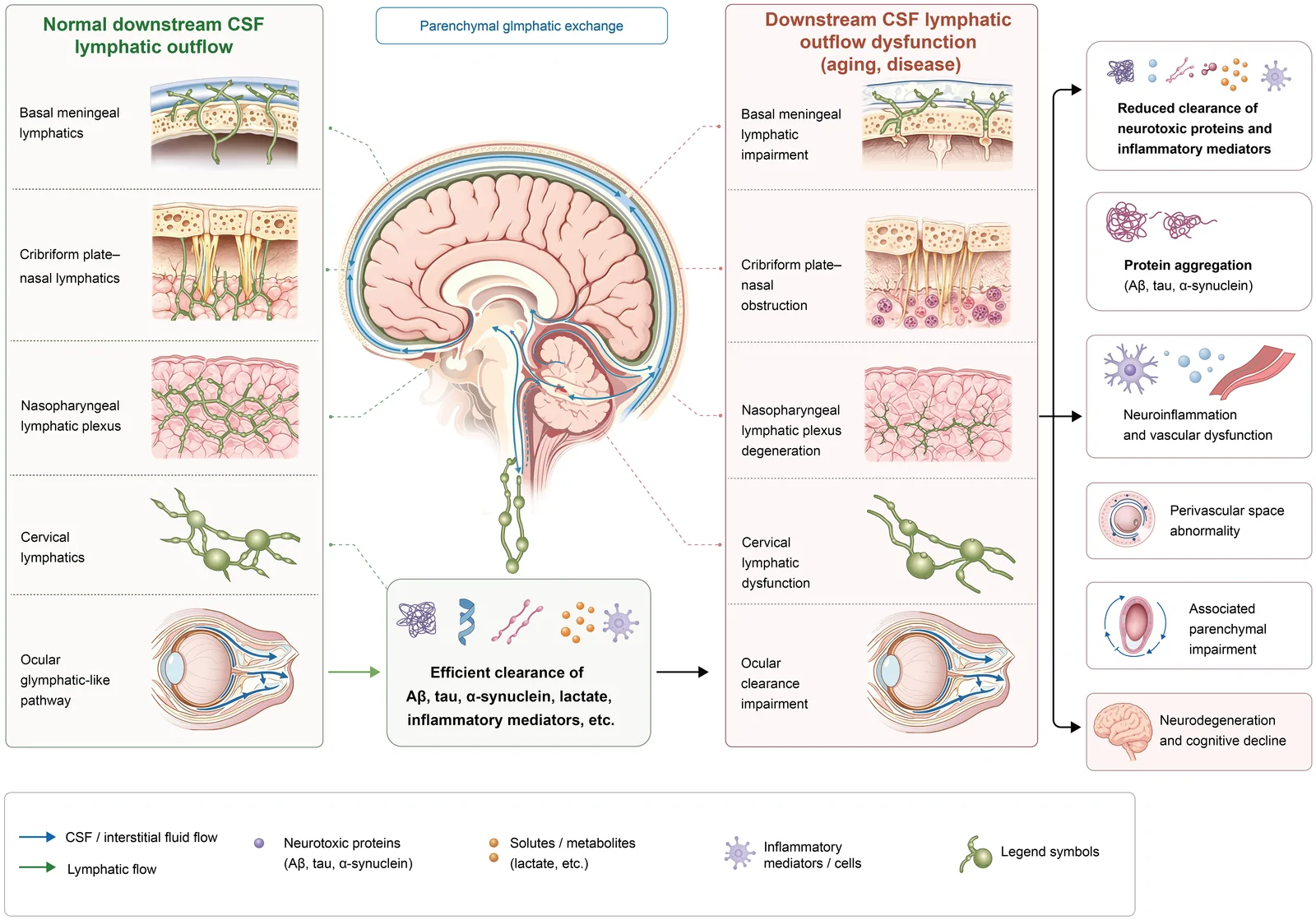
Integrated dysfunction of parenchymal glymphatic exchange and downstream CSF lymphatic outflow. The model distinguishes parenchymal glymphatic exchange from anatomically downstream CSF lymphatic outflow within the broader brain-fluid clearance network. Under physiological conditions, CSF–ISF exchange and perivascular solute movement occur within the brain parenchyma, whereas subsequent fluid and solute transport may involve basal meningeal lymphatic vessels, the cribriform plate–nasal lymphatic pathway, the nasopharyngeal lymphatic plexus and cervical lymphatic vessels. The ocular glymphatic-like pathway is presented as a related but parallel fluid-transport hypothesis rather than as an established component of cerebral CSF or protein clearance. Evidence for these routes is derived largely from bulk-fluid or generic CSF tracer studies and should not be equated with cargo-specific clearance. Evidence for soluble-protein clearance, aggregated-protein handling and immune-antigen drainage varies amongst cargoes and anatomical routes; therefore, the cargoes shown do not imply that each has been directly demonstrated to traverse every depicted pathway. During ageing or disease, dysfunction of one or more downstream routes may impair fluid transport and may be associated with impaired parenchymal glymphatic exchange, protein accumulation, neuroinflammation and neurodegeneration. The model therefore presents these processes as interacting but anatomically distinct. This image is an original schematic created by the authors using Adobe Illustrator and was not adapted or reproduced from any previously published source.

## Standardised definitions and biomarkers

A first priority is to establish consistent terminology and operational definitions. Parenchymal CSF–ISF exchange, perivascular solute movement, meningeal lymphatic uptake, skull-base egress, nasopharyngeal drainage, cervical lymphatic transport, and ocular glymphatic-like movement should not be treated as interchangeable processes. Studies should specify which anatomical component is being measured and whether the outcome reflects structural anatomy, bulk-fluid or generic-tracer movement, clearance of a defined soluble protein, handling of aggregated proteins, drainage of CNS-derived antigens or inflammatory molecules, lymphatic uptake, or downstream transport. Evidence in one category should not be treated as proof of another, and findings for one cargo or anatomical route should not be generalised to other cargoes or routes without direct experimental support.

Biomarkers should be validated against clearly defined biological processes. DTI-ALPS, olfactory testing, ophthalmic imaging, and fluid biomarkers may provide complementary information, but none should be regarded as a direct or specific measure of CSF outflow in isolation. Standardised acquisition protocols, analytical methods, anatomical regions of interest, and reporting criteria will be required to improve reproducibility across studies.

## Human imaging and functional validation

Direct assessment of skull-base, meningeal, nasopharyngeal, cervical, and ocular fluid-transport pathways remains a major challenge in humans. Future studies should combine high-resolution anatomical imaging with dynamic or functional measurements capable of evaluating transport direction, timing, and efficiency.

Multimodal approaches may integrate MRI-based measures, lymphatic imaging, fluid biomarkers, ophthalmic assessments, and clinical outcomes. However, structural visualisation of a vessel or pathway does not demonstrate effective drainage, and tracer distribution does not necessarily indicate physiological clearance. Human studies must therefore distinguish vessel anatomy, fluid movement, pathological leakage, and effective removal of solutes.

## Longitudinal studies of causality

Most available human evidence is cross-sectional and cannot determine whether altered outflow precedes neurodegeneration, develops secondary to tissue injury, or reflects shared effects of ageing, vascular dysfunction, inflammation, and sleep disturbance.

Longitudinal studies in at-risk populations are therefore required. Relevant cohorts may include individuals with genetic or biomarker-defined risk of Alzheimer’s disease, mild cognitive impairment, isolated REM sleep behaviour disorder, cerebral small-vessel disease, early glaucoma, or age-related retinal disease. Repeated assessment of outflow-related measures, protein biomarkers, neuroinflammation, imaging abnormalities, and cognitive or visual outcomes may help determine whether alterations in specific pathways predict subsequent disease progression.

## Interventional target-engagement studies

Candidate interventions should first demonstrate biological target engagement before claims of disease modification are made. Studies of VEGF-C–VEGFR3 signalling, cervical lymphatic contractility, nitric oxide or adrenergic modulation, mechanical stimulation, AQP4-associated water transport, pressure gradients, and pupillary responses should establish whether the intervention directly changes the intended transport pathway.

Increased tracer movement alone is insufficient. Interventional studies should determine whether target engagement improves solute clearance, modifies Aβ, tau, α-synuclein, or inflammatory biomarkers, preserves neural structure, and produces meaningful cognitive, neurological, or visual outcomes. Disease stage is also likely to influence therapeutic response, because improving fluid transport may have limited benefit after protein aggregation, vascular injury, synaptic dysfunction, and neuroinflammation have become self-sustaining.

## Safety considerations

Safety should be assessed with the same rigour as efficacy. Enhanced lymphatic drainage should not be assumed to be uniformly beneficial, because increased transport may facilitate the removal of inflammatory material but may also increase antigen delivery to cervical lymph nodes and amplify peripheral immune responses. These effects are likely to be disease-specific and stage-dependent. Manipulating CSF or lymphatic transport may alter intracranial or intraocular pressure, worsen oedema during ischaemia or barrier disruption, modify immune-cell trafficking, redistribute inflammatory mediators, promote maladaptive lymphangiogenesis, or influence the spread of infection through nasal or cervical lymphatic routes. AQP4 modulation may also produce opposing effects depending on tissue oedema, barrier integrity, anatomical location and disease stage. Interventional studies should therefore define anatomical specificity, dose, timing, reversibility and long-term effects before clinical translation.

## Cross-species translational challenges

Cross-species anatomical and physiological differences substantially limit direct extrapolation of rodent findings to humans. The morphology of the cribriform plate and olfactory apparatus varies considerably across mammals, including differences in cribriform plate surface area, foraminal organisation and the anatomical scale of the olfactory system (92). In mice, tracer studies have directly demonstrated CSF passage through the cribriform plate along olfactory nerve-associated routes into nasal submucosal lymphatics (11). However, these findings do not establish that the transcribriform route makes an equivalent quantitative contribution to CSF outflow in humans. Accordingly, the prominent contribution of olfactory mucosal lymphatics observed in rodents should not be assumed to represent human physiology without direct functional validation.

Skull-base and meningeal lymphatic structures show partially conserved anatomical organisation across rodents, non-human primates and humans (1). Similarly, a nasopharyngeal lymphatic plexus has been identified in both mice and macaques; however, its valve morphology differs between species, and functional CSF tracer drainage through this pathway has been demonstrated predominantly in mice (12). Therefore, anatomical conservation does not necessarily imply equivalent transport capacity, regulation or contribution to total CSF outflow across species. The quantitative role of basal meningeal, cribriform and nasopharyngeal lymphatic pathways in living humans remains unresolved.

The optic nerve lamina also differs substantially across species. Humans and larger mammals possess a collagenous lamina cribrosa composed of connective-tissue beams that partition the neural tissue into pores, whereas the analogous mouse glial lamina lacks a comparable collagenous beam network and is organised predominantly around astrocytic structures (93). These differences may influence translaminar pressure transmission, barrier properties, tracer distribution and susceptibility to glaucoma-related injury. Rodent ocular studies should therefore be interpreted as mechanistic evidence for possible transport processes rather than as direct quantitative estimates of human ocular glymphatic-like physiology.

## Conclusion

Brain fluid clearance comprises anatomically connected but functionally distinct processes, including parenchymal CSF–ISF exchange, perivascular solute transport, meningeal and skull-base lymphatic uptake, and downstream extracranial drainage. Current evidence supports the cribriform–olfactory route, basal meningeal lymphatics, the nasopharyngeal lymphatic plexus, cervical lymphatic vessels and the eye–optic nerve axis as potential components of this broader clearance network. The skull base and nasopharyngeal region may represent important interfaces between intracranial CSF and extracranial lymphatic drainage, although their quantitative contribution to human CSF outflow remains unresolved.

Available clinical measures provide indirect and biologically distinct information. DTI-ALPS reflects directional diffusivity along presumed perivascular-space regions rather than direct CSF flow or extracranial outflow; olfactory dysfunction is nonspecific; and abnormal ocular tracer movement may reflect altered transport, pathological leakage or barrier disruption. These measures should therefore not be interpreted individually as direct evidence of impaired CSF clearance.

Overall, alterations in CSF outflow may occur as consequences, modifiers or contributors to neurodegenerative disease, but their causal and therapeutic significance remains unproven. Establishing their clinical relevance will require route-specific definitions, validated human measurements, longitudinal evidence and interventional studies demonstrating both biological target engagement and meaningful clinical effects. Until such evidence is available, CSF outflow pathways should be regarded as mechanistically plausible but incompletely validated components of neurodegenerative disease biology.
